# The Critical Role of PARPs in Regulating Innate Immune Responses

**DOI:** 10.3389/fimmu.2021.712556

**Published:** 2021-07-22

**Authors:** Huifang Zhu, Yan-Dong Tang, Guoqing Zhan, Chenhe Su, Chunfu Zheng

**Affiliations:** ^1^ Neonatal/Pediatric Intensive Care Unit, Children’s Medical Center, First Affiliated Hospital of Gannan Medical University, Ganzhou, China; ^2^ State Key Laboratory of Veterinary Biotechnology, Harbin Veterinary Research Institute of Chinese Academy of Agricultural Sciences, Harbin, China; ^3^ Department of Infectious Disease, Renmin Hospital, Hubei University of Medicine, Shiyan, China; ^4^ The Wistar Institute, Philadelphia, PA, United States; ^5^ Department of Microbiology, Immunology and Infectious Diseases, University of Calgary, Calgary, AB, Canada

**Keywords:** PARP, ADP-ribosylation, innate immune responses, inflammation, NLR (NOD-like receptor)

## Abstract

Poly (adenosine diphosphate-ribose) polymerases (PARPs) are a family of proteins responsible for transferring ADP-ribose groups to target proteins to initiate the ADP-ribosylation, a highly conserved and fundamental post-translational modification in all organisms. PARPs play important roles in various cellular functions, including regulating chromatin structure, transcription, replication, recombination, and DNA repair. Several studies have recently converged on the widespread involvement of PARPs and ADP-Ribosylation reaction in mammalian innate immunity. Here, we provide an overview of the emerging roles of PARPs family and ADP-ribosylation in regulating the host’s innate immune responses involved in cancers, pathogenic infections, and inflammations, which will help discover and design new molecular targets for cancers, pathogenic infections, and inflammations.

## Introduction

Innate immunity is an evolutionary conservative defense system that generates prompt immune responses to protect the host from tumorigeneses and pathogenic infections, which depends on the recognition of endogenous damage-associated molecular patterns (DAMPs) or exogenous conserved pathogen-associated molecular patterns (PAMPs) in many microorganisms by host pattern recognition receptors (PRRs), including four main families: Toll-like receptors (TLRs), retinoic acid-inducible gene I (RIG-I)-like receptors (RLRs), cytosolic DNA sensor cyclic GMP-AMP synthase (cGAS)-stimulator of interferon genes (STING) axis, and the nucleotide-binding oligomerization domain (NOD)-like receptors (NLRs). Innate immunity is characterized by the production of inflammatory mediators and/or type I interferons (IFN-I) and thousands of IFN-induced genes (ISGs).

ADP-ribosylation is catalyzed by ADP-ribosyl transferases (ARTs) through transferring ADP-ribose groups from nicotinamide adenine dinucleotide (NAD^+^) to their target proteins. PARPs are the best-understood ARTs, and to date, 17 members of PARPs in humans have been identified. Based on the characteristics of their structural and functional domains, PARPs are broadly categorized as four subfamilies: DNA-dependent PARPs (including PARP1, PARP2, and PARP3), Tankyrases PARPs (including PARP5a and PARP5b), Macrodomain-containing PARPs (including PARP9, PARP14, and PARP15), and CCCH (Cys-Cys-Cys-His) zinc finger-containing PARPs (including PARP7, PARP12, and PARP13) (1). Besides, the Trp-Trp-Glu motif (WWE) domain is another important regulatory domain sharing in some PARPs, including PARP7, PARP11, PARP12, PARP13, and PARP14. In contrast, others fall outside of these groups as their domains are either unique (PARP4 and PARP16) or uncharacterized (PARP6 and PARP8) (2). According to their catalytic activities, PARPs are categorized as poly-ADP-ribose polymerases, mono-ADP-ribose polymerases, and inactive ADP-ribose polymerases. Mono ADP-ribosylation (MARylation) is initiated by the PARP monoenzymes, like PARP3, PARP6, PARP10, PARP14, PARP15, and PARP16, to catalyze the addition of a single ADP-ribose unit to amino acids; while poly-ADP-ribosylation (PARylation) is carried out by the PARP polymerases, like PARP1, PARP2, PARP5a, and PARP5b, to transfer of multiple units of ADP-ribose on target proteins. Emerging data shows that PARPs are broadly involved in various cellular processes, especially in DNA repair and transcription, exampled as PARP1, PARP2, and PARP3. DNA damage caused by replication stresses or free radical formation is deleterious for maintaining genome integrity; therefore, mammalian cells have evolved a signaling pathway called DNA damage response (DDR) to protect against genomic insults. DNA damage is normally recognized and repaired by the intrinsic DDR machinery. In response to DNA damage, PARP1 detects and binds to the break sites and ensures other repair factors initiating the DNA repair processes. In addition to the DDR, PARPs also function during other cell stress responses such as unfolded protein response (UPR), stress granule (SG) formation, and pathogenic infections (3). The Malik lab’s evolutionary analyses indicate that most PARP genes’ rapid evolution is closely correlated with the pathogen defense proteins, suggesting a close connection between the antiviral responses and PARPs activities (4). Several PARPs, like PARP4, 7, 9, 12, 13, and 14, were reported to possess broad-spectrum antiviral activities (5–10). PARPs also have broad regulatory effects in promoting or suppressing inflammatory activation. This review will focus on the emerging role of PARPs in regulating the innate immune responses associated with antitumor, antiviral, and inflammatory responses.

## PARPs in Regulating DDRs and cGAS-STING-Mediated Anti-Tumorigenic Innate Immune Responses

DNA damage events caused by intra- and inter-cellular stimuli occur in our bodies’ cells every minute of the day, greatly threatening their genetic integrity. Cells have evolved multiple mechanisms, such as transient cell cycle arrest and DNA repair responses, to maintain genomic stability. The DNA double-strand break (DSB) is the most cytotoxic form of DNA damage, leading to the accumulation of DNA fragments in the cytoplasm, termed cytoplasmic chromatin fragments (CCF), which can be recognized by the nucleic acid sensor cyclic cGAS (11, 12). After binding with exogenous segments of base-paired DNA, cGAS experiences a conformational change and enables cyclic GMP-AMP (cGAMP) synthesis, which subsequently activates adaptor protein STING and recruits TANK-binding kinase 1 (TBK1) and the heterodimeric IKK α/β (inhibitor kappa B kinase α/β) kinase for phosphorylation, and then promotes the activation of interferon regulatory factor 3 (IRF3) and nuclear factor kappa-light-chain-enhancer of activated B cells (NF-κB), respectively. IRF3 and NF-κB act as transcription factors in the nucleus to trigger the transcription of IFN-I. Besides, some DNA damaging agents and ionizing irradiation are reported to induce IFN-I production through the cGAS-cGAMP-STING-mediated innate immune signaling pathway and promote antitumor immunity (13, 14), and cellular responses to DNA damage are important determinants of cancer development and outcome following radiation therapy and chemotherapy.

**Figure 1:**
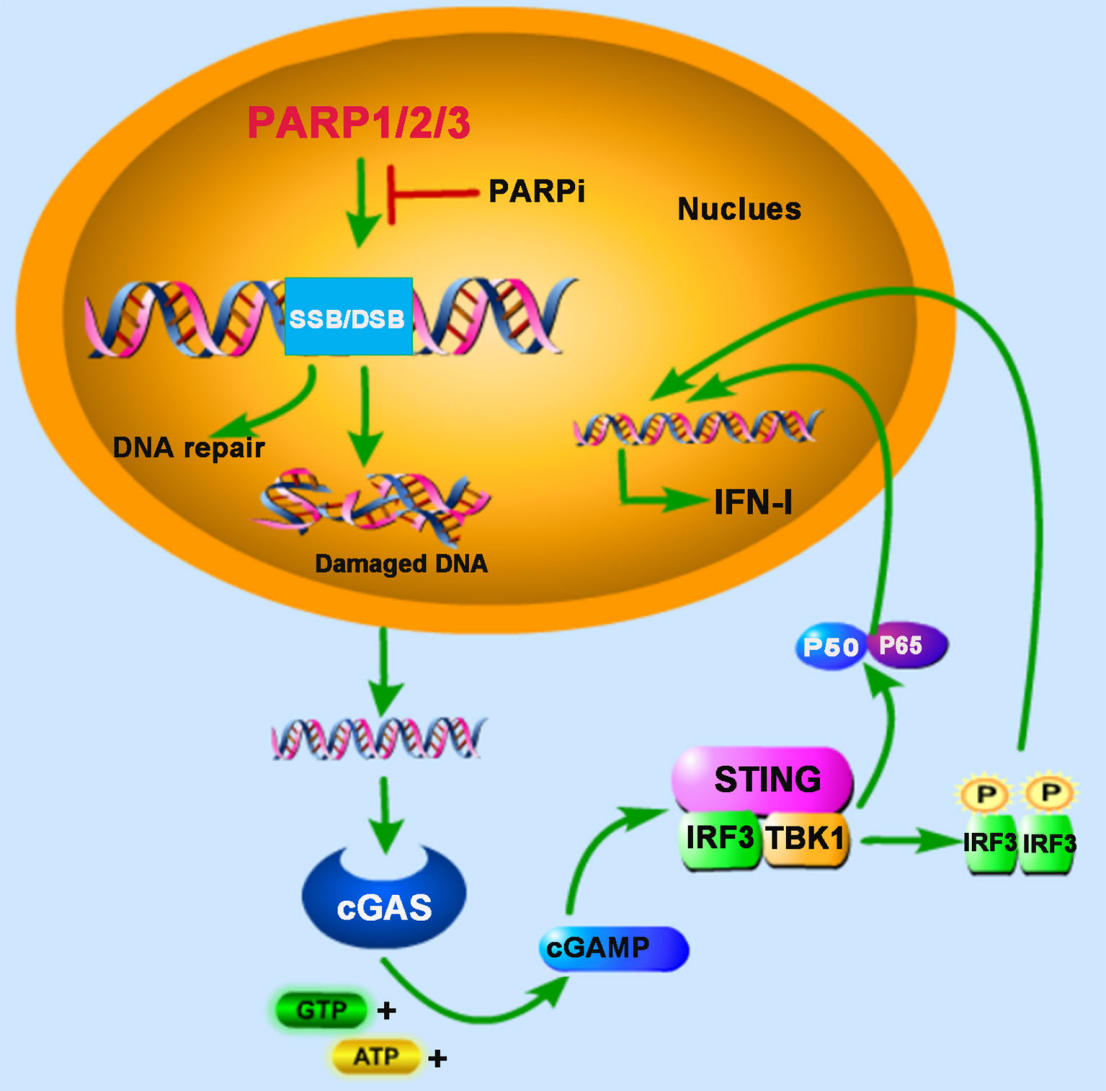
PARPs regulate cGAS-STING-mediated innate immune responses. PARP1, PARP2, and PARP3 participate in the DNA repair process upon the DNA damage response (DDR) caused by DNA double-strand breaks (DSB) or DNA single-strand breaks (SSB). The treatment of PARP inhibitor (PARPi) prevents DNA repair, resulting in the accumulation of damaged DNA, which is recognized by cyclic GMP-AMP (cGAMP) synthase (cGAS) in the cytoplasm. The induction of type I interferon (IFN-I) is mediated by cAMP-STING-IRF3/NF-κB signaling pathway.

PARP1 and PARP2 are localized in the nucleus and share approximately 69% homology in their catalytic domains. Both are involved in the early DNA damage response to participate in the DNA repair process (15, 16). In some advanced tumors, the DDR pathway’s targeting is demonstrated to be an attractive potential therapeutic strategy (17). Furthermore, PARP inhibitor (PARPi) has received some encouraging results in clinical trials for the treatment of cancers, for example, breast cancer susceptibility gene (BRCA)-deficient ovarian and breast cancers (18, 19). BRCA proteins are involved in repairing DNA DSBs. In BRCA-deficient cells, DNA repair mediated by PARP1 is essential for cell survival. PARPi blocks PARP1’s function and inevitably generates cytosolic DNA in BRCA mutant cancer cells, which triggers the cGAS-STING signaling pathway (20) (**Figure 1**). In human cancer cell lines (HeLa, UPN251, and HOC1), PARPi treatment leads to cytosolic DNA accumulation and activates downstream signaling molecules TBK1 and IRF3. Knockdown of cGAS or STING reduces the immune responses resulting from PARPi treatment, indicating that PARPi-triggered activation of antitumor immunity is mediated by the cGAS-STING signaling pathway (21). These findings strongly support the involvement of PARPs in the crosstalk between innate immunity and cancer biology.

## PARPs in Regulating RNA Sensors-Mediated Antiviral Immune Responses

Viral infection recognition by host innate immunity is the first and the most important event to eliminate viruses. PRRs are a group of proteins employed by the innate immune system to recognize PAMPs or DAMPs to evoke the host’s first-line defense. The recognition function of PRRs is usually activated by special PAMPs from pathogens such as viral glycoproteins and nucleic acids. Recently, PARPs are demonstrated to be involved in regulating antiviral innate immune responses during the virus-host interactions (4, 22). RIG-I is a cytosolic DExD/H box RNA helicase that belongs to the RLR family, which contains an RNA helicase domain and two N-terminal caspase activation and recruitment domain (CARD) domains responsible for the transduction of signal to downstream signaling adaptor mitochondrial antiviral-signaling protein (MAVS) upon detection of viral double-stranded RNA (dsRNA) (23). Subsequently, the activated MAVS recruits downstream proteins TBK1 and/IKKϵ, which phosphorylate and activate IRF3 and IRF7, stimulating IFN-I production with the help of other transcription factors. Accumulating evidence reveals that several members of PARPs show a particular effect on the host antiviral defense during viral infection. PARP13, also called ZAP (Zinc-Finger Antiviral Protein), was the first identified and the most studied PARP family member with antiviral activity. It can inhibit the replication and infection of several viruses by participating in multiple cellular pathways. Two recent studies from Sindbis virus (SINV) and Japanese encephalitis virus (JEV) infected cell models, respectively, demonstrate that ZAP could enhance the antiviral innate immune responses associate with the RIG-I signaling pathway (24, 25).

**Figure 2:**
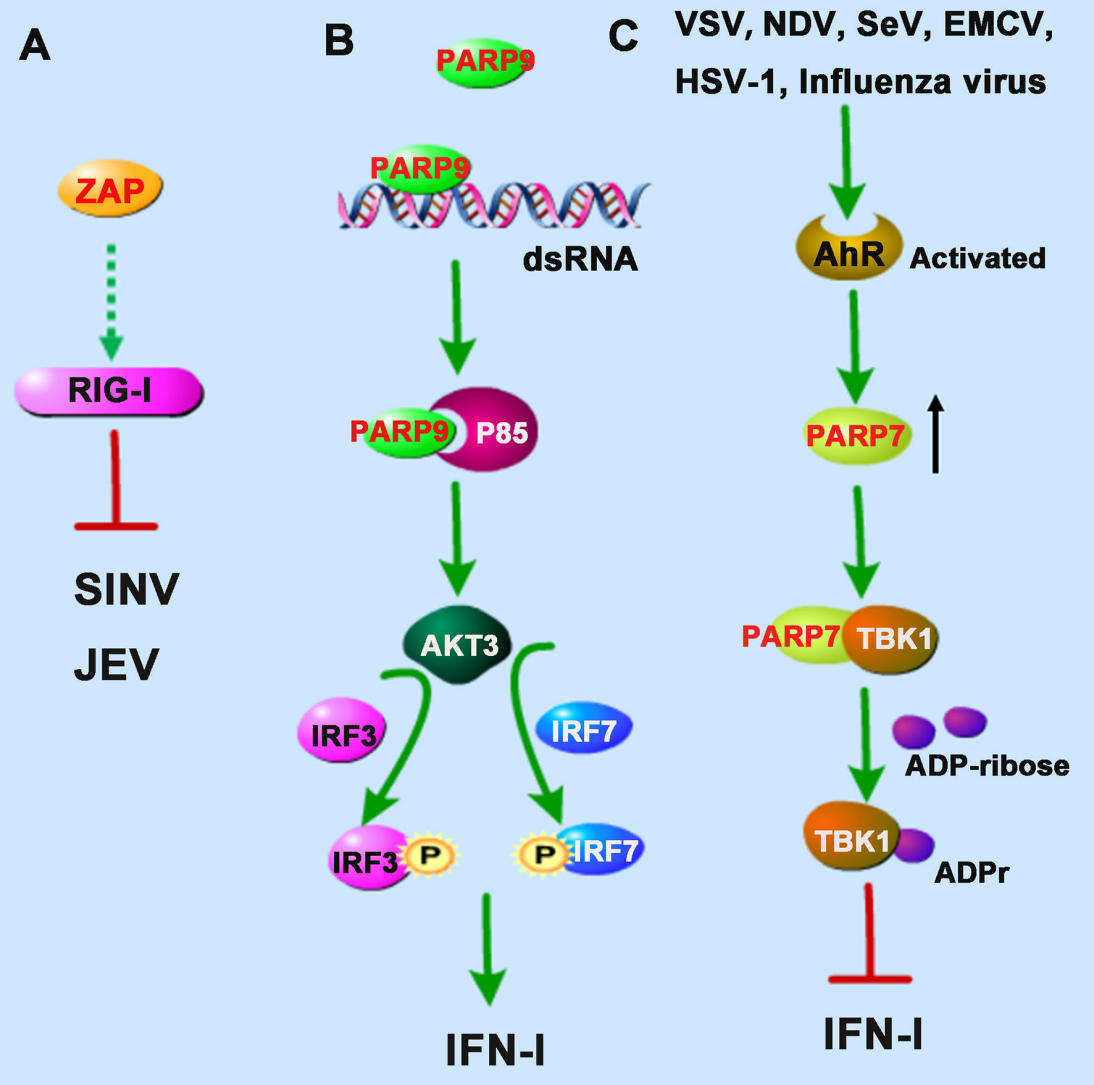
PARPs regulate the RNA sensors-mediated antiviral innate immune responses. **(A)** ZAP inhibits Sindbis virus (SINV) and Japanese encephalitis virus (JEV) probably by enhancing the RIG-I signaling pathway. **(B)** PARP9 recognizes and binds to dsRNA from reovirus or poly(I:C) and subsequently binds to and activates the PI3K regulatory subunit p85, and then further triggers the downstream AKT3 activation to phosphorylate IRF3 and IRF7 for IFN-I production. **(C)** The infection of vesicular stomatitis virus (VSV), influenza virus, Newcastle disease virus (NDV), Sendai virus (SeV), encephalomyocarditis virus (EMCV), or herpes simplex virus-1 (HSV-1) could activate the aryl hydrocarbon receptor (AhR), which subsequently upregulates the expression of PARP7. PARP7 physically interacts with TBK1 and inhibits its activity by ADP-ribosylation, abolishing the antiviral IFN-I signaling pathway. Dashed lines indicate uncertain interactions or that the underlying mechanism is not known.

Interestingly, TRIM25, an E3 ubiquitin ligase responsible for the poly-ubiquitination and activation of RIG-I, was also found to catalyze K48- and K63-linked polyubiquitination of ZAP during SINV infection in 293T cells (24). Although the ubiquitination of ZAP seemed not required for its antiviral activity, the interaction with TRIM25 could greatly promote ZAP’s antiviral activity. However, it is not clear whether the antiviral role of ZAP is dependent on the RIG-I signaling pathway (**Figure 2A**). The researchers hypothesized that TRIM25 might also ubiquitinate some other signal molecules complexed with ZAP during viral infection (24).

Intriguingly, a recent study identified PARP9 as a noncanonical sensor for RNA virus in dendritic cells during the IFN-I-mediated antiviral process. PARP9 could recognize and bind to viral genomic dsRNA from reovirus or dsRNA poly(I:C), and its antiviral role relied on the activation of the phosphoinositide 3-kinase (PI3K)/AKT3 pathway, but not the MAVS signaling pathway, to produce IFN-I (26). PI3K is composed of a catalytic subunit called p110 and a regulatory subunit named p85. Mechanistically, PARP9 could bind to and activate the regulatory subunit p85, which further triggered the downstream AKT3 activation to phosphorylate IRF3 and IRF7 for IFN-I production (**Figure 2B**).

In addition to participation in the positively regulating antiviral responses, some PARP family members also show potentiality to regulate the production of IFN-I negatively. The aryl hydrocarbon receptor (AhR) is a cytoplasmic receptor/transcription factor modulating several cellular and immunological processes following viral infections. A recent study showed that upon the infection of mouse hepatitis virus strain A59 (MHV-A59), a prototypical coronavirus, in bone marrow-derived macrophages (BMDMs), AhR is activated and contributes to the upregulation of PARP7. Knockdown of PARP7 reduces viral replication and increases IFN expression, suggesting that PARP7 functions as a pro-viral factor during MHV infection (27). However, the regulatory mechanism still needed further investigation.

Similarly, results from another group also demonstrated that the expression of PARP7 was upregulated following activation of the AhR. While in AhR-deficient MEF cells, upon the infection of vesicular stomatitis virus (VSV), influenza virus, Newcastle disease virus (NDV), Sendai virus (SeV), encephalomyocarditis virus (EMCV), or herpes simplex virus-1 (HSV-1), the antiviral IFN-I responses were stronger than that of wild-type MEF cells (28). The underlying mechanism is that PARP7 could physically interact with TBK1 and inhibit its activity by ADP-ribosylation, abolishing the antiviral IFN-I signaling pathway (28), which revealed the physiological importance of endogenous AhR signal activation involving in innate responses mediated by IFN-I, suggesting that the AhR-PARP7 axis is a potential therapeutic target for enhancing antiviral immune responses (**Figure 2C**). The exact positioning of the ADP-ribosylation site of TBK1 needs to be further clarified. Although the activity of TBK1 is regulated by multiple pathways, such as phosphorylation and ubiquitination (29), there are very few reports of ADP-ribosylation of TBK1. Taken together, we speculate that the catalytic activity of PARP7 can be used as a therapeutic target for regulating TBK1 kinase activity to control inflammation and cancer.

In addition to the PARPs discussed above, more PARPs are involved in the antiviral innate responses. Our recently published review (30) has specifically summarized them in detail; therefore, we will not restate them here.

## PARPs in Regulating NF-κB-Mediated Inflammatory Responses

Inflammation is an essential immune response that protects the body from pathogenic infections, toxic stimulations, or autoimmune damages. Accordingly, untimely controlled destruction will increase pro-inflammatory mediators, lead to persistent tissue damage, and promote various chronic diseases, including diabetes, cancer, autoimmune diseases, and inflammatory bowel diseases. Therefore, a full understanding of inflammatory responses’ regulatory mechanisms will provide novel insights to treat a range of inflammation-associated diseases.

Evidence from bacterial lipopolysaccharide (LPS) stimulated monocytes/macrophages indicates that ADP-ribosylation participates in inflammation. The induction of inflammatory cytokines tumor necrosis factor-α (TNF-α), IL-1, and IL-6 by LPS were attenuated by PARP inhibitor, indicating that PARPs may play major roles in promoting inflammatory responses through the expression of pro-inflammatory cytokines (31, 32). Since then, the link between PARP activity and inflammation has been studied extensively. PARP1-deficient mice provided evidence that PARP1 promotes NF-κB activation in macrophages *in vivo* (33). This study demonstrated that PARP1 deletion leads to a marked resistance to LPS-induced endotoxic shock resulted from inducible nitric oxide synthase (iNOS) and NO through the NF-κB signaling pathway (33). A recent study suggests that phosphorylation of PARP1 results in the PARylation of the NF-κB subunit p65/RelA, which induces the transcription of NF-κB-regulated genes (34). However, in some cases, neither the enzymatic activity nor the DNA binding activity seems to be required for PARP1 to induce the inflammatory responses-mediated by NF-κB, but interestingly, the direct interaction with both subunits of NF-κB (p50 and p65) by PARP1 is required for the production of NF-κB-inducible cytokines, indicating PARP1 as an essential and novel classical transcriptional coactivator for kappa B-dependent gene expression (35). In macrophages, PARP1 also induces the release of the high-mobility group box 1 (HMGB1), a pro-inflammatory factor, from the nucleus to the cytoplasm, which requires its PARylation and subsequent acetylation (36, 37). In other macrophage-related cell types, such as Kupffer cells (KCs) during chronic alcoholic liver injury, or microglia in the injured brain, PARP1 also exerts pro-inflammatory effects (38, 39).

**Figure 3:**
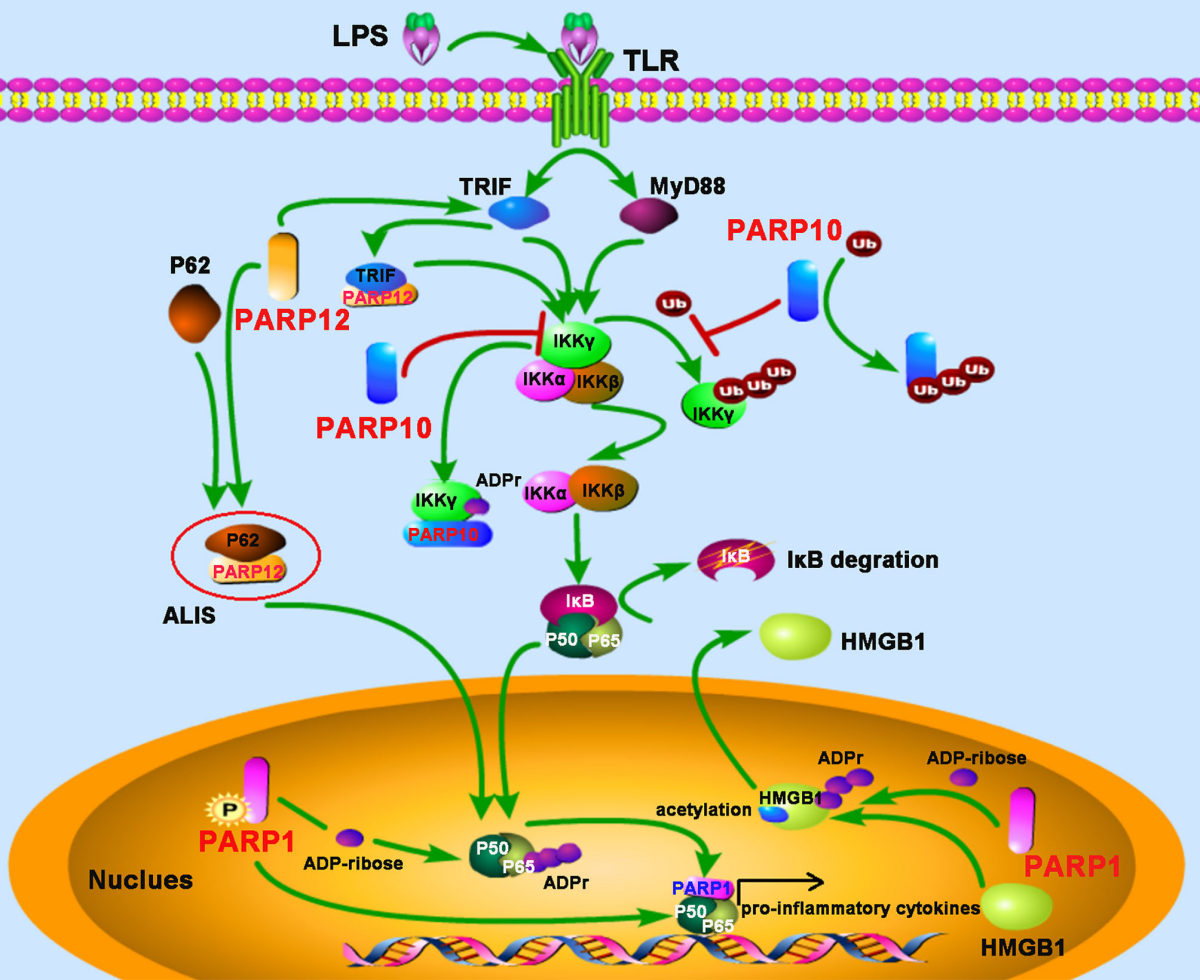
PARPs regulate the NF-κB-mediated inflammatory responses. LPS stimulates the activation of the NF-κB signaling pathway depending on TRIF (toll-receptor-associated activator of interferon) and MyD88 (myeloid differentiation factor 88). PARP12 is recruited and co-localized with p62 in the aggresome-like induced structures (ALIS), which acts as a platform for NF-κB signaling; PARP12 interacts and co-localizes with TRIF, leading to an enhancement of NF-κB signaling. PARP10 inhibits the activation of IKKγ through two mechanisms: competitively binding of K63-polyubiquitination to interfere with IKKγ’s polyubiquitylation and interacting with IKKγ to promote its mono-ADP-ribosylation, leading to the inhibition of p65 translocation into the nucleus. The phosphorylation of PARP1 results in the PARylation of the NF-κB subunit p65. The direct interaction with both subunits of NF-κB (p50 and p65) by PARP1 promotes NF-κB-inducible cytokine production. PARP1 mediates the PARylation of high-mobility group box 1 (HMGB1), following by subsequent acetylation, and then induces its release from the nucleus to the cytoplasm.

NEMO (NF-κB essential modifier), also called IKKγ, is required for the activation of the IκB kinase complex by inflammatory stimuli such as TNF-α (40). The polyubiquitylation of NEMO is essential for the canonical NF-κB pathway (41). PARP10, also known as ARTD10, prevents the K63-polyubiquitination of NEMO by ADP-ribosylating NEMO, which negatively regulates the NF-κB signaling pathway (42). The underlying mechanism is that the UIM (ubiquitin interaction motif) domain of PARP10 can bind to K63-linked polyubiquitin and interfere with NEMO’s polyubiquitylation, leading to the inhibition of p65 translocation into the nucleus. Further study also demonstrates that PARP10 interacts with NEMO and promotes its mono-ADP-ribosylation, a novel post-translational modification implicated in NF-κB signaling (42). SQSTM1, also named p62, is an adaptor and scaffold protein involved in cell death, inflammation signaling, and autophagy. Upon LPS stimulation in RAW264.7 macrophages, PARP12 was recruited and co-localized with p62/SQSTM1 in the aggresome-like induced structures (ALIS), which acted as a platform for NF-κB signaling. Further study suggested that ectopic expression of PARP12 could interact and co-localize with the toll-receptor-associated activator of interferon (TRIF), an important kinase involved in NF-κB activation, leading to an enhancement of NF-κB signaling and secretion of IL-8, suggesting a role for PARP12 in inflammation (43) (**Figure 3**).

## PARPs in Regulating Stress Responses-Mediated Innate Immune Responses

**Figure 4:**
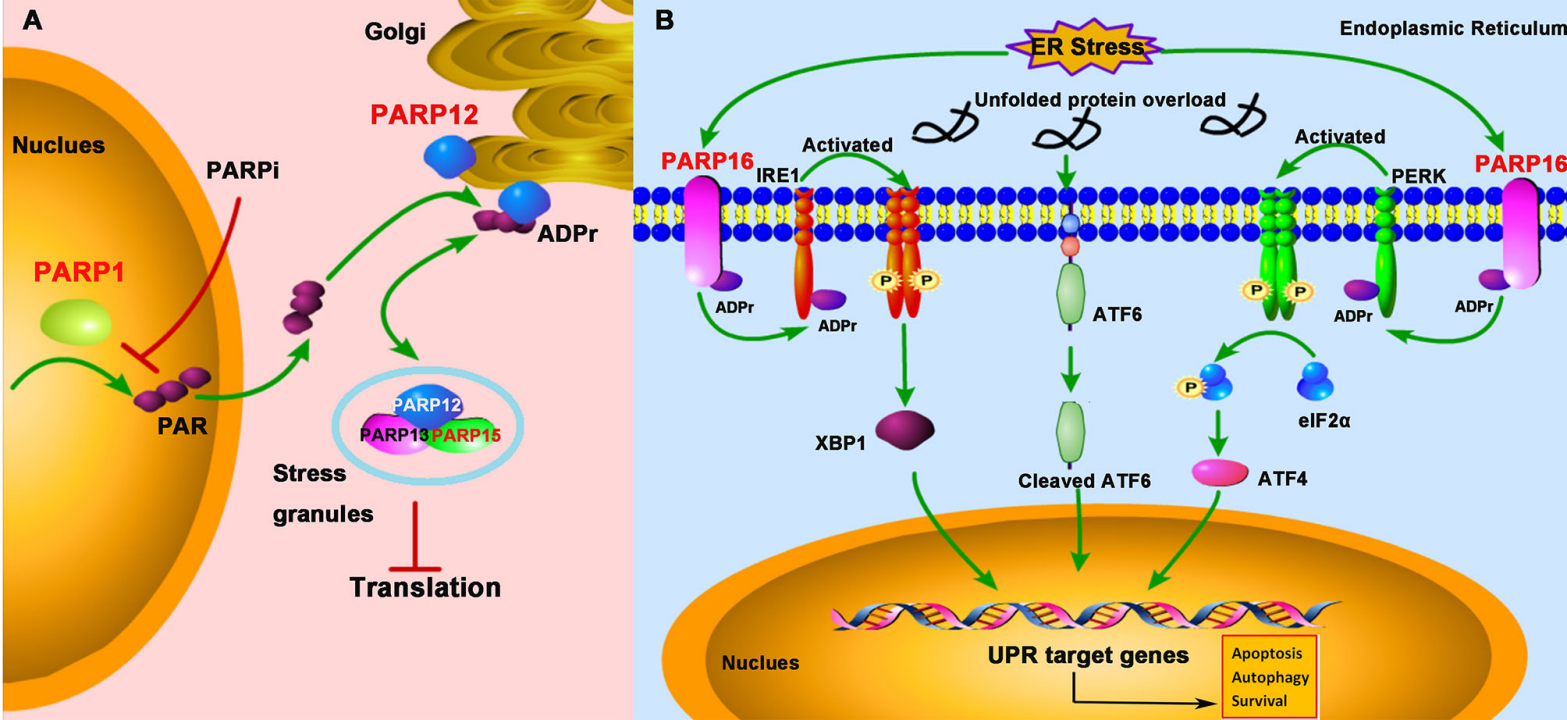
PARPs regulate the cellular stress-mediated innate immune responses. **(A)** PARP1 catalyzes the formation and release of poly-ADP-ribose (PAR) from the nucleus; subsequently, PAR binds to PARP12 and promotes its translocation from the Golgi to stress granule (SG), where PARP12, PARP13, and PARP15 form a complex to block the protein translation. The treatment of PARPi against PARP1 prevents PAR formation and PARP12 translocation from the Golgi to SG. **(B)** The unfolded protein response (UPR) activation is initiated by three ER (endoplasmic reticulum)-localized integral membrane proteins, PKR-like endoplasmic reticulum kinase (PERK), Activating Transcription Factor 6 (ATF6), and Inositol-Requiring Enzyme 1 (IRE1). PARP16 localizes in the ER and is upregulated during ER stress, which subsequently promotes the mono-ADP-ribosylation (ADPr) of IRE1 and PERK. ADP-ribosylated IRE1 and PERK are activated by phosphorylation and form dimers in the ER.

Cells are exposed to diverse stresses, including genotoxic, oxidative, and pathogenic infections. In addition to DDR, PARPs regulate other cellular stress responses, such as SG formation and UPR (**Figure 4**).

## SG

SGs are non-membrane-bound organelles localized in the cytoplasm. They are aggregates of untranslated messenger ribonucleoproteins (mRNPs) complex to stall the translation initiation (44). The SG formation is broadly linked to cellular stress responses and viral infections and is a novel concept in the antiviral innate immunity (45). X-ray crystallography data demonstrated that PARP13 and other functional partners PARP12 and PARP15 were localized in SG (46). The localization to SG is important for PARP13 to exhibit its antiviral activity against alphaviruses (47). Besides, under the cellular stress challenge, PARP12, which is initially localized in the Golgi, translocates from the Golgi complex to cytoplasmic SG. Mechanistically, PARP1 catalyzes poly-ADP-ribose (PAR) formation, which then is released from the nucleus and subsequently binds to the PARP12 WWE domain, resulting in the translocation of PARP12 from the Golgi to SG. On the other hand, inhibition of PARP1-mediated PAR formation prevents PARP12 translocation to SG (48). The translocation of PARP12 to SG is reversible, as it relocates back to the Golgi once the stress is relieved (**Figure 4A**).

## UPR

The UPR is a conserved stress response activated when misfolded proteins accumulate in the endoplasmic reticulum (ER), a protective mechanism for cells to maintain cellular homeostasis in response to ER stress. Once the protein homeostasis is broken, UPR activation is initiated by three ER-localized integral membrane proteins, PKR-like Endoplasmic Reticulum Kinase (PERK), Activating Transcription Factor 6 (ATF6), and Inositol-Requiring Enzyme 1 (IRE1), and will promote cell death through apoptosis or autophagy pathway (49). Accumulating evidence suggests that some human diseases, like cancers, inflammations, and viral infections, are associated with ER stress (50). Some viruses such as herpesviruses and coronavirus have evolved to hijack the UPR signaling to facilitate viral replication (51, 52). Therefore, a growing interest focuses on targeting the UPR signaling pathway as a therapeutic strategy against viral infections. PARP16 contains an α-helical domain and acts as mono-ADP-ribose polymerases. It was showed that PARP16 is a novel tail-anchored ER protein required for the PERK- and IRE1-mediated unfolded protein response (53). It was upregulated during ER stress and was required for the UPR (54, 55) (**Figure 4B**). When knockdown of PARP16 expression, cells are highly sensitive to ER stress, resulting in increased cell death (53), suggesting that PARP16 could be an attractive therapeutic inhibition target for cancers, viral infections, and inflammations.

## Conclusions

The diverse roles of PARPs implicated in various human diseases have been widely complimented by recent advances that link ADP-ribosylation to stress responses, metabolisms, viral infections, and cancers, in which the PARPs are predicated to be potent therapeutical targets, especially that cancer treatment with PARP inhibitors has received some considerable success. Moreover, recent studies revealed that some members of PARPs are also involved in host-virus interactions. Recent advance implicates the PARPs family as potent antiviral regulators, which would be a promising topic. The current pandemic of coronavirus disease 2019 (COVID-2019), caused by the severe acute respiratory syndrome Coronavirus (SARS-CoV-2), still poses a threat to global public health. Enormous efforts have been devoted to this virus. Recently, a study used MHV as a model coronavirus demonstrated that in human lung cell lines, the infection of SARS-CoV-2 strikingly upregulated the noncanonical MARylating PARP isozymes including PARP7, PARP9, PARP10, PARP11, PARP12, PARP13, and PARP14, and then depressed the cellular NAD metabolome by inducing the expression of enzymes for salvage NAD synthesis from nicotinamide (NAM) and nicotinamide riboside (NR), and reducing other NAD biosynthetic (56). Given that the availability of NAD could limit the antiviral activity of PARPs upon coronaviruses, modulating the NAD activity may be a novel strategy to restrict viral infection potentially.

Furthermore, the latest finding in the recognization of SARS-CoV-2 with a low abundance of CpG dinucleotides by PARP13 (57) may subvert our previous understanding that CpG-rich viral genomic RNA would be a favorite for its recognition. Although SARS-CoV-2 shows low CpG frequencies throughout most parts of its genome, the number of CpGs at the 3’ end is higher, which may render SARS-CoV-2 still sensitive to PARP13 restriction. As an ISG gene, PARP13 was demonstrated to be induced by all three types of IFNs and reduce viral RNA in human epithelial lung cancer cell lines Calu-3 and A549. Interestingly, IFN-γ seemed to be the most effective IFN to induce PARP13 and inhibit SARS-CoV-2; however, the underlying mechanism is still unknown (57). The antiviral roles of PARPs may have been far underappreciated, and more investigations are needed in the future. The antiviral effects of some PARPs and the immune evasion mechanisms by viruses will bring new insights into the treatment of viral infectious diseases. For example, the coronavirus macrodomain can prevent PARP14-mediated IFN-I production and promote viral replication, indicating that the macrodomain may be a potent target for antiviral therapy (10).

Meanwhile, agonists for PARP14 and some other antiviral PARPs seem to be workable by enhancing the host’s IFN-I-mediated antiviral responses. Nevertheless, beyond acting as antiviral regulators, PARPs are involved in multiple cellular activities, especially those that play an important role in promoting inflammation. Therefore, it should be cautious about developing a target therapeutic strategy associated with PARPs regulated innate immune signaling. Furthermore, separation-of-function targeting of PARPs will achieve tailored therapeutic effects. Much more studies are encouraged to reveal the defense mechanisms by PARPs.

## Author Contributions

HZ, Y-DT, GZ, and CS write the draft. CZ review and supervise the manuscript. All authors contributed to the article and approved the submitted version.

## Funding

This article is funded by the National Natural Science Foundation of China (82003043), and the Natural Science Foundation of Jiangxi Province (20202BAB216002).

## Conflict of Interest

The authors declare that the research was conducted in the absence of any commercial or financial relationships that could be construed as a potential conflict of interest.
