# Corrigendum: The critical role of PARPs in regulating innate immune responses

**DOI:** 10.3389/fimmu.2023.1253094

**Published:** 2023-07-18

**Authors:** Huifang Zhu, Yan-Dong Tang, Guoqing Zhan, Chenhe Su, Chunfu Zheng

**Affiliations:** ^1^ Neonatal/Pediatric Intensive Care Unit, Children’s Medical Center, First Affiliated Hospital of Gannan Medical University, Ganzhou, China; ^2^ State Key Laboratory of Veterinary Biotechnology, Harbin Veterinary Research Institute of Chinese Academy of Agricultural Sciences, Harbin, China; ^3^ Department of Infectious Disease, Renmin Hospital, Hubei University of Medicine, Shiyan, China; ^4^ The Wistar Institute, Philadelphia, PA, United States; ^5^ Department of Microbiology, Immunology and Infectious Diseases, University of Calgary, Calgary, AB, Canada

**Keywords:** ADP-ribosylation (ADPRylation), NLR (NOD-like receptor), PARP (poly(ADP-ribose) polymerase, inflammation, innate immune responses

In the published article, there was an error in the affiliations. Instead of six, there are only five affiliations. The original affiliation 2 has been removed. The correct affiliations are listed below:

Instead of

Huifang Zhu ^1^,^2^, Yan-Dong Tang ^3^, Guoqing Zhan ^4^, Chenhe Su ^5^, Chunfu Zheng ^2^, ^6^

“^1^Neonatal/Pediatric Intensive Care Unit, Children’s Medical Center, First Affiliated Hospital of Gannan Medical University, Ganzhou, China.

^2^Department of Immunology, School of Basic Medical Sciences, Fujian Medical University, Fuzhou, China.

^3^State Key Laboratory of Veterinary Biotechnology, Harbin Veterinary Research Institute of Chinese Academy of Agricultural Sciences, Harbin, China.

^4^Department of Infectious Disease, Renmin Hospital, Hubei University of Medicine, Shiyan, China.

^5^The Wistar Institute, Philadelphia, PA, United States.

^6^Department of Microbiology, Immunology and Infectious Diseases, University of Calgary, Calgary, AB, Canada.”,

it should be

Huifang Zhu ^1^, Yan-Dong Tang ^2^, Guoqing Zhan ^3^, Chenhe Su ^4^, Chunfu Zheng ^5^

“^1^Neonatal/Pediatric Intensive Care Unit, Children’s Medical Center, First Affiliated Hospital of Gannan Medical University, Ganzhou, China.

^2^State Key Laboratory of Veterinary Biotechnology, Harbin Veterinary Research Institute of Chinese Academy of Agricultural Sciences, Harbin, China.

^3^Department of Infectious Disease, Renmin Hospital, Hubei University of Medicine, Shiyan, China.

^4^ The Wistar Institute, Philadelphia, PA, United States.

^5^Department of Microbiology, Immunology and Infectious Diseases, University of Calgary, Calgary, AB, Canada.”

The authors apologize for this error and state that this does not change the scientific conclusions of the article in any way. The original article has been updated.

